# Fault Detection and Diagnosis for Gas Turbines Based on a Kernelized Information Entropy Model

**DOI:** 10.1155/2014/617162

**Published:** 2014-08-28

**Authors:** Weiying Wang, Zhiqiang Xu, Rui Tang, Shuying Li, Wei Wu

**Affiliations:** ^1^College of Power and Energy Engineering, Harbin Engineering University, Harbin 150001, China; ^2^Harbin Marine Boiler & Turbine Research Institute, Harbin 150036, China; ^3^Harbin Institute of Technology, Harbin 150001, China

## Abstract

Gas turbines are considered as one kind of the most important devices in power engineering and have been widely used in power generation, airplanes, and naval ships and also in oil drilling platforms. However, they are monitored without man on duty in the most cases. It is highly desirable to develop techniques and systems to remotely monitor their conditions and analyze their faults. In this work, we introduce a remote system for online condition monitoring and fault diagnosis of gas turbine on offshore oil well drilling platforms based on a kernelized information entropy model. Shannon information entropy is generalized for measuring the uniformity of exhaust temperatures, which reflect the overall states of the gas paths of gas turbine. In addition, we also extend the entropy to compute the information quantity of features in kernel spaces, which help to select the informative features for a certain recognition task. Finally, we introduce the information entropy based decision tree algorithm to extract rules from fault samples. The experiments on some real-world data show the effectiveness of the proposed algorithms.

## 1. Introduction

Gas turbines, mechanical systems operating on a thermodynamic cycle, usually with air as the working fluid, are considered as one kind of the most important devices in power engineering, where the air is compressed, mixed with fuel, and burnt in a combustor, with the generated hot gas expanded through a turbine to generate power, which is used for driving the compressor and for providing the means to overcome external loads. Gas turbines play an increasingly important role in the domains of mechanical drives in the oil and gas sectors, electricity generation in the power sector, and propulsion systems in the aerospace and marine sectors.

Safety and economy are always two fundamentally important factors in designing, producing, and operating gas turbine systems. Once a malfunction occurs to a gas turbine, a serious accident, even disaster, may take place. It was reported that about 25 accidents take place every year due to jet malfunctioning. In 1989, 111 were killed in a plane crash due to an engine fault. Although great progress has been made these years in the area of condition monitoring and fault diagnosis, how to predict and detect malfunctions is still an open problem for the complex systems. In some cases, such as offshore oil well drilling platforms, the main power system is self-monitoring without man on duty. So the reliability and stabilization are of critical importance to these systems. There are hundreds of offshore platforms with gas turbines providing electricity and powers in China. There is an urgent requirement to design and develop online remote monitoring and health management techniques for these systems.

More than two hundred sensors are installed in each gas turbine for monitoring the state of a gas turbine. The data gathered by these sensors reflects the state and trend of the system. If we build a center to monitor two hundred gas turbine systems, we should watch the data coming from more than forty thousand sensors. Obviously, it is infeasible to manually analyze them. Techniques on intelligent data analysis have been employed in gas turbine monitoring and diagnosis. In 2007, Wang et al. designed a conceptual system for remote monitoring and fault diagnosis of gas turbine-based power generation systems [1]. In 2008, Donat et al. discussed the issue of data visualization, data reduction, and ensemble learning for intelligent fault diagnosis in gas turbine engines [2]. In 2009, Li and Nilkitsaranont described a prognostic approach to estimating the remaining useful life of gas turbine engines before their next major overhaul based on a combined regression technique with both linear and quadratic models [3]. In the same year, Bassily et al. proposed a technique, which assessed whether or not the multivariate autocovariance functions of two independently sampled signals coincide, to detect faults in a gas turbine [4]. In 2010, Young et al. presented an offline fault diagnosis method for industrial gas turbines in a steady-state using Bayesian data analysis. The authors employed multiple Bayesian models via model averaging for improving the performance of the resulted system [5]. In 2011, Yu et al. designed a sensor fault diagnosis technique for Micro-Gas Turbine Engine based on wavelet entropy, where wavelet decomposition was utilized to decompose the signal in different scales, and then the instantaneous wavelet energy entropy and instantaneous wavelet singular entropy are computed based on the previous wavelet entropy theory [6].

In recent years, signal processing and data mining techniques are combined to extract knowledge and build models for fault diagnosis. In 2012, Wu et al. studied the issue of bearing fault diagnosis based on multiscale permutation entropy and support vector machine [7]. In 2013, they designed a technique for defecting diagnostics based on multiscale analysis and support vector machines [8]. Nozari et al. presented a model-based robust fault detection and isolation method with a hybrid structure, where time-delay multilayer perceptron models, local linear neurofuzzy models, and linear model tree were used in the system [9]. Sarkar et al. [10] designed symbolic dynamic filtering by optimally partitioning sensor observation, and the objective is to reduce the effects of sensor noise level variation and magnify the system fault signatures. Feature extraction and pattern classification are used for fault detection in aircraft gas turbine engines.

Entropy is a fundamental concept in the domains of information theory and thermodynamics. It was first defined to be a measure of progressing towards thermodynamic equilibrium; then it was introduced in information theory by Shannon [11] as a measure of the amount of information that is missing before reception. This concept gets popular in both domains [12–16]. Now it is widely used in machine learning and data driven modeling [17, 18]. In 2011, a new measurement, called maximal information coefficient, was reported. This function can be used to discover the association between two random variables [19]. However, it cannot be used to compute the relevance between feature sets.

In this work, we will develop techniques to detect abnormality and analyze faults based on a generalized information entropy model. Moreover, we also describe a system for state monitoring of gas turbines on offshore oil well drilling platforms. First we will describe a system developed for remote and online condition monitoring and fault diagnosis of gas turbines installed on oil drilling platforms. As vast amount of historical records is gathered in this system, it is an urgent task to design algorithms for automatically online detecting abnormality of the data and analyze the data to obtain the causes and sources of faults. Due to the complexity of gas turbine systems, we focus on the gas-path subsystem in this work. The function of entropy is employed to measure the uniformity of exhaust temperatures, which is a key factor reflecting the health of the gas path of a gas turbine and also reflecting the performance of the gas turbine. Then we extract features from the healthy and abnormal records. An extended information entropy model is introduced to evaluate the quality of these features for selecting informative attributes. Finally, the selected features are used to build models for automatic fault recognition, where support vector machines [20] and C4.5 are considered. Real-world data are collected to show the effectiveness of the proposed techniques.

The remainder of the work is organized as follows.  Section 2 describes the architecture of the remote monitoring and fault diagnosis center for gas turbines installed on the oil drilling platforms.  Section 3 designs an algorithm for detecting abnormality of the exhaust temperatures. Then we extract features from the exhaust temperature data and select informative ones based on evaluating the information bottlenecks with extend information entropy in Section 4. Support vector machines and C4.5 are introduced for building fault diagnosis models in Section 5. In addition, numerical experiments are also described in this section. Finally, conclusions and future work are given in Section 6.

## 2. Framework of Remote Monitoring and Fault Diagnosis Center for Gas Turbine

**Figure 1:**
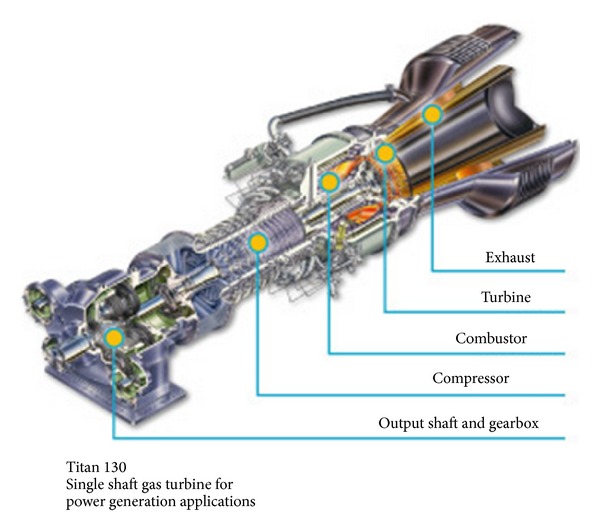
Prototype structure of a gas turbine.

Gas turbines are widely used as power and electric power sources. The structure of a general gas turbine is presented in Figure 1. This system transforms chemical energy into thermal power, then mechanical energy, and finally electric energy. Gas turbines are usually considered as the hearts of a lot of mechanical systems.

As the offshore oil well drilling platforms are usually unattended, an online and remote state monitoring system is much useful in this area, which can help find abnormality before serious faults occur. However, the sensor data cannot be sent into a center with ground based internet. The data can only be transmitted via telecommunication satellite, which was too expensive in the past. Now this is available.

**Figure 2:**
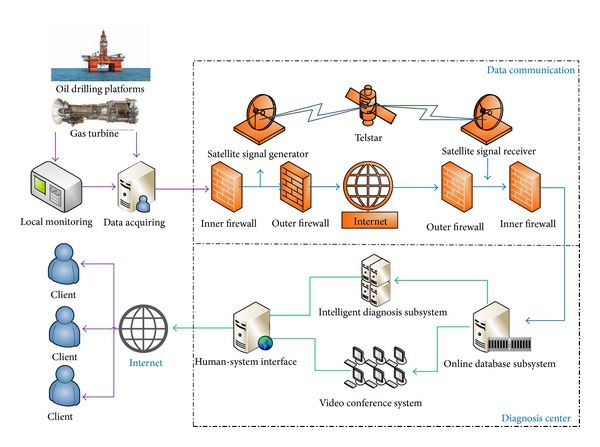
Structure of the remote system of condition monitoring and fault analysis.

The system consists of four subsystems: data acquisition and local monitoring subsystem (DALM), data communication subsystem (DAC), data management subsystem (DMS), and intelligent diagnosis system (IDS). The first subsystem gathers the outputs from different sensors and checks whether there is any abnormality in the system. The second one packs the acquired data and transforms them into the monitoring center. Users in the center can also send a message to this subsystem to ask for some special data if abnormality or fault occurs. The data management subsystem stores the historic information and also fault data and fault cases. A data compression algorithm is embedded in the system. As most of the historic data are useless for the final analysis, they will be compressed and removed for saving storage space. Finally, IDS watches the alarm information from different unit assemblies and starts the corresponding module to analyze the related information. This system gives some decision and explains how the decision has been made. The structure of the system is shown in Figure 2.

**Figure 3:**
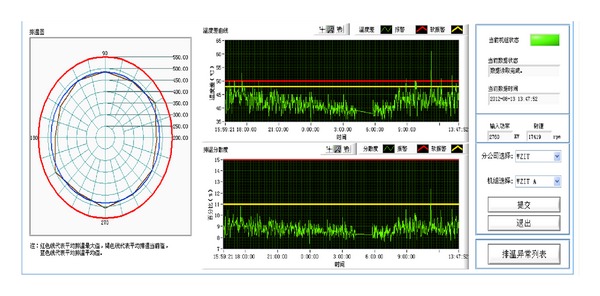
A typical webpage for monitoring of the subsystem.

One of the webpages of the system is given in Figure 3, where we can see the rose figure of exhaust temperatures, and some statistical parameters varying with time are also presented.

## 3. Abnormality Detection in Exhaust Temperatures Based on Information Entropy

Exhaust temperature is one of the most critical parameters in a gas turbine as excessive turbine temperatures may lead to life reduction or catastrophic failures. In the current generation of machines, temperatures at the combustor discharge are too high for the type of instrumentation available. Exhaust temperature is also used as an indicator of turbine inlet temperature.

As the temperature profile out of a gas turbine is not uniform, a number of probes will help pinpoint disturbances or malfunctions in the gas turbine by highlighting the shifts in the temperature profile. Thus there are usually a set of thermometers fixed on the exhaust. If the system is normally operating, all the thermometers give similar outputs. However, if a fault occurs to some components of the turbine, different temperatures will be observed. The uniformity of exhaust temperatures reflects the state of the system. So we should develop an index to measure the uniformity of the exhaust temperatures. In this work, we consider the entropy function for it is widely used in measuring uniformity of random variables. However, to the best of our knowledge, this function has not been used in this domain.

Assume that there are *n* thermometers and their outputs are *T*_*i*_, *i* = 1,…, *n*, respectively. Then we define the uniformity of these outputs as

(1) $$\begin{array}{c} E(T)=-\overset{n}{\underset{i=1}{\sum }}\frac{T_{i}}{T}\log_{2}\frac{T_{i}}{T}, \end{array}$$

where *T* = ∑_*j*_ *T*_*j*_. As *T*_*i*_ ≥ 0, we define 0log⁡0 = 0.

Obviously, we have log⁡_2_ *n* ≥ *E*(*T*) ≥ 0. *E*(*T*) = log⁡_2_ *n* if and only if *T*_1_ = *T*_2_ = ⋯ = *T*_*n*_. In this case, all the thermometers produce the same output. So the uniformity of the sensors is maximal. In another extreme case, if *T*_1_ = *T*_2_ = *T*_*i*−1_ = *T*_*i*+1_ ⋯ = *T*_*n*_ = 0 and *T*_*i*_ = *T*, then *E*(*T*) = 0.

It is notable that the value of entropy is independent of the values of thermometers, while it depends on the distribution of the temperatures. The entropy is maximal if all the thermometers output the same values.

Now we show two sets of real exhaust temperatures measured on an oil well drilling platform, where 13 thermometers are fixed. In the first set, the gas turbine starts from a time point and then runs for several minutes; finally the system stops.

**Figure 4:**
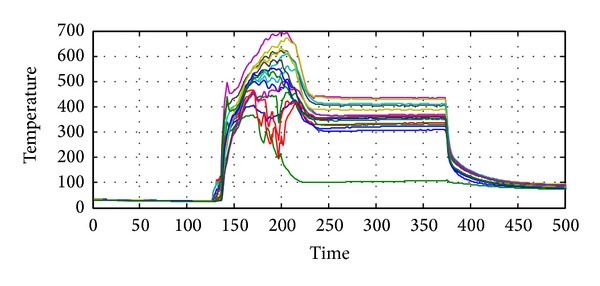
Exhaust temperatures from a set of thermometers.

Observing the curves in Figure 4, we can see that the 13 thermometers give the almost the same outputs at the beginning. In fact, the outputs are the room temperature in this case, as shown in Figure 6(a). Thus, the entropy reaches the peak value.

**Figure 5:**
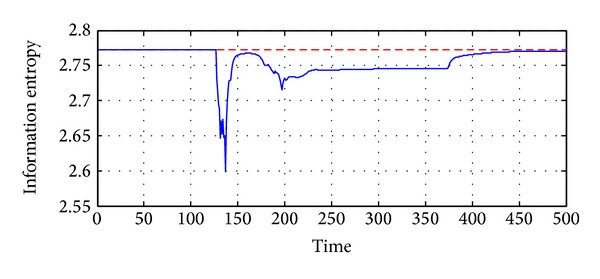
Uniformity of the temperatures (red dash line is the ideal case; blue line is the real case).

**Figure 6:**
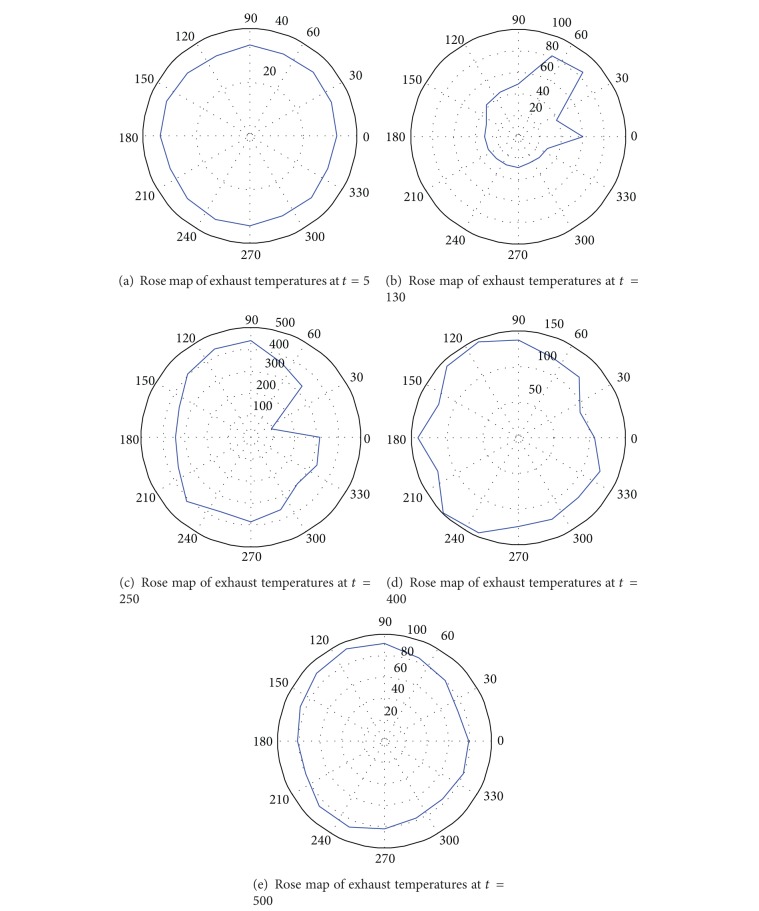
Samples of temperature distribution in different times.

Some typical samples are presented in Figure 6, where the temperature distributions around the exhaust at time points *t* = 5,130,250,400, and 500 are given. Obviously, the distributions at *t* = 130,250, and 400 are not desirable. It can be derived that some abnormality occurs to the system. The entropy of temperature distribution is given in Figure 5.

**Figure 7:**
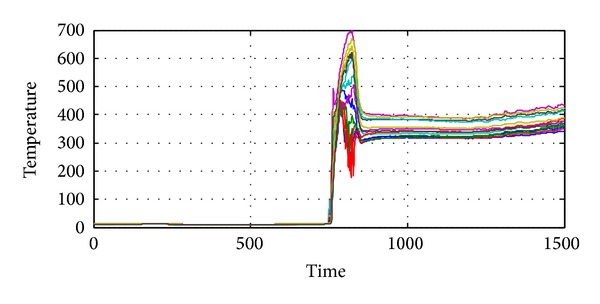
Exhaust temperatures from another set of thermometers.

**Figure 8:**
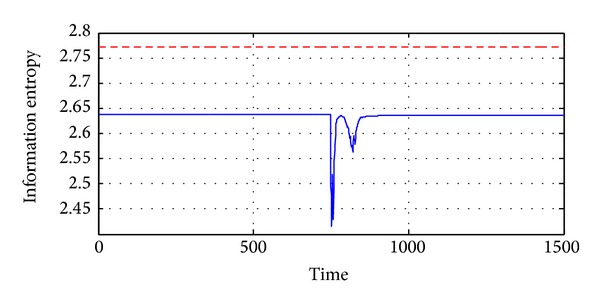
Entropy of the temperature distribution, where the red dash line is the ideal case and the blue one is the real case.

**Figure 9:**
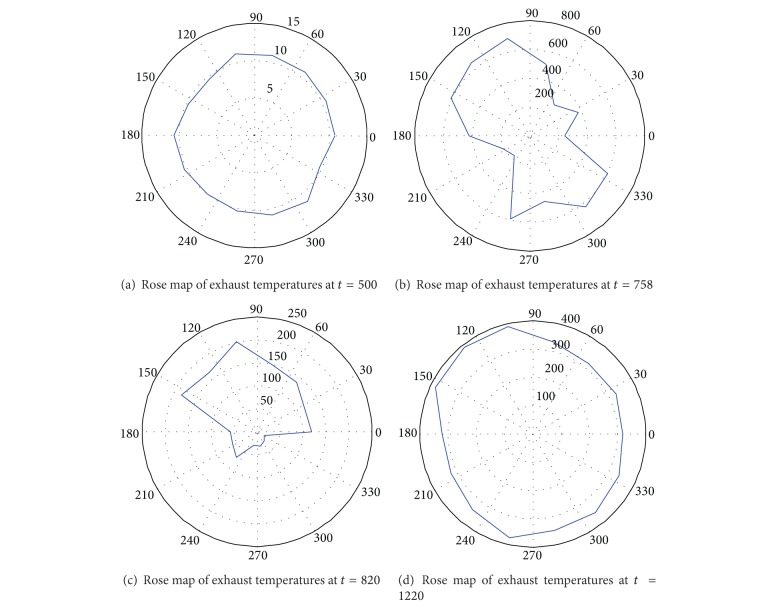
Samples of temperature distribution in different moments.

Another example is also given in Figures 7 to 9. In this example, there is significant difference between the outputs of 13 thermometers even when the gas turbine is not running, just as shown in Figure 9(a). Thus the entropy of temperature distribution is a little lower than the ideal case, as shown in Figure 8. Besides, some representative samples are also given in Figure 9.

Considering the above examples, we can see that the function of entropy is an effective measurement of uniformity. It can be used to reflect the uniformity of exhaust temperatures. If the uniformity is less than a threshold, some faults possibly occur to the gas path of the gas turbine. Thus the entropy function is used as an index of the health of the gas path.

## 4. Fault Feature Quality Evaluation with Generalized Entropy

The above section gives an approach to detecting the abnormality in the exhaust temperature distribution. However, the function of entropy cannot distinguish what kind of faults occurs to the system although it detects abnormality. In order to analyze why the temperature distribution is not uniform, we should develop some algorithms to recognize the fault.

Before training an intelligent model, we should construct some features and select the most informative subsets to represent different faults. In this section, we will discuss this issue.

Intuitively, we know that the temperatures of all thermometers reflect the state of the system. Besides, the temperature difference between neighboring thermometers also indicates the source of faults, which are considered as space neighboring information. Moreover, we know the temperature change of a thermometer necessarily gives hints to study the faults, which can be viewed as time neighboring information. In fact, the inlet temperature *T*_0_ is also an important factor. In summary, we can use exhaust temperatures and their neighboring information along time and space to recognize different faults. If there are *n* (*n* = 13 in our system) thermometers, we can form a feature vector to describe the state of the exhaust system as

(2) F=T0,T1,T2,…,Tn,T1−T2,T2−T3,…,Tn−T1,T1′,T2′,…,Tn′,

where *T*_*i*_′ = *T*_*i*_(*j*) − *T*_*i*_(*j* − 1). *T*_*i*_(*j*) is the temperature at time *j* of the *i*th thermometer.

Apart from the above features, we can also construct other attributes to reflect the conditions of the gas turbine. In this work, we consider a gas turbine with 13 thermometers around the exhaust. So we can form a 40-attribute vector finally.

There are some questions whether all the extracted features are useful for final modeling and how we can evaluate the features and find the most informative features. In fact, there are a number of measures to estimate feature quality, such as dependency in the rough set theory [21], consistency [22], mutual information in the information theory [23], and classification margin in the statistical learning theory [24]. However, all these measures are computed in the original input space, while the effective classification techniques usually implement a nonlinear mapping of the original space to a feature space by a kernel function. In this case, we require a new measure to reflect the classification information of the feature space. Now we extend the traditional information entropy to measure it.

Given a set of samples *U* = {*x*_1_, *x*_2_,…, *x*_*m*_}, each sample is described with *n* features *F* = {*f*_1_, *f*_2_,…, *f*_*n*_}. As to classification learning, each training sample *x*_*i*_ is associated with a decision *y*_*i*_. As to an arbitrary subset *F*′⊆*F* and a kernel function *K*, we can calculate a kernel matrix

(3) $$\begin{array}{c} K=\left[\begin{array}{ccc} k_{11} & \ldots & k_{1m}\\ \vdots & \ddots & \vdots \\ k_{m1} & \ldots & k_{mm} \end{array}\right], \end{array}$$

where *k*_*ij*_ = *k*(*x*_*i*_, *x*_*j*_). The Gaussian function is a representative kernel function:

(4) $$\begin{array}{c} k_{ij}=\exp(-\frac{{x_{i}-x_{j})}^{2}}{\sigma }). \end{array}$$

A number of kernel functions have the properties (1)  *k*_*ij*_ ∈ [0,1]; (2)  *k*_*ij*_ = *k*_*ji*_.

Kernel matrix plays a bottleneck role in kernel based learning [25]. All the information that a classification algorithm can use is hidden in this matrix. In the same time, we can also calculate a decision kernel matrix as

(5) $$\begin{array}{c} D=\left[\begin{array}{ccc} d_{11} & \ldots & d_{1m}\\ \vdots & \ddots & \vdots \\ d_{m1} & \ldots & d_{mm} \end{array}\right], \end{array}$$

where *d*_*ij*_ = 1 if *y*_*i*_ = *y*_*j*_; otherwise, *d*_*ij*_ = 0. In fact, the matrix *D* is a matching kernel.

**Definition 1 .**

Given a set of samples *U* = {*x*_1_, *x*_2_,…, *x*_*m*_}, each sample is described with *n* features *F* = {*f*_1_, *f*_2_,…, *f*_*n*_}. *F*′⊆*F*, *K* is a kernel matrix over *U* in terms of *F*′. Then the entropy of *F*′ is defined as

(6) $$\begin{array}{c} E(K)=-\frac{1}{m}\overset{m}{\underset{i=1}{\sum }}\log_{2}\frac{K_{i}}{m}, \end{array}$$

where *K*_*i*_ = ∑_*j*=1_^*m*^ *k*_*ij*_.

As to the above entropy function, if we use Gaussian function as the kernel, we have log⁡_2_ *m* ≥ *E*(*K*) ≥ 0. *E*(*K*) = 0 if and only if *k*_*ij*_ = 1  ∀*i*, *j*. *E*(*K*( = log⁡_2_ *m* if and only if *k*_*ij*_ = 0, *i* ≠ *j*. *E*(*K*) = 0 means that any pair of samples cannot be distinguished with the current features, while *E*(*K*) = log⁡_2_ *m* means any pair of samples is different from each other. So they can be distinguished. These are two extreme cases. In real-world applications, part of samples can be discerned with the available features, while others are not. In this case, the entropy function takes value in the interval [0, log⁡_2_ *m*].

Moreover, it is easy to show that if *K*_1_⊆*K*_2_, *E*(*K*_1_) ≥ *E*(*K*_2_), where *K*_1_⊆*K*_2_ means *K*_1_(*x*_*i*_, *x*_*j*_) ≤ *K*_2_(*x*_*i*_, *x*_*j*_), ∀*i*, *j*.

**Definition 2 .**

Given a set of samples *U* = {*x*_1_, *x*_2_,…, *x*_*m*_}, each sample is described with *n* features *F* = {*f*_1_, *f*_2_,…, *f*_*n*_}. *F*_1_, *F*_2_⊆*F*. *K*_1_ and *K*_2_ are two kernel matrices induced by *F*_1_ and *F*_2_. *K* is a new function computed with *F*_1_ ∪ *F*_2_. Then the joint entropy of *F*_1_ and *F*_2_ is defined as

(7) $$\begin{array}{c} E(K_{1},K_{2})=E(K)=-\frac{1}{m}\overset{m}{\underset{i=1}{\sum }}\log_{2}\frac{K_{i}}{m}, \end{array}$$

where *K*_*i*_ = ∑_*j*=1_^*m*^ *k*_*ij*_.

As to the Gaussian function, *K*(*x*_*i*_, *x*_*j*_) = *K*_1_(*x*_*i*_, *x*_*j*_) × *K*_2_(*x*_*i*_, *x*_*j*_). Thus *K*⊆*K*_1_ and *K*⊆*K*_2_. In this case, *E*(*K*) ≥ *E*(*K*_1_) and *E*(*K*) ≥ *E*(*K*_2_).

**Definition 3 .**

Given a set of samples *U* = {*x*_1_, *x*_2_,…, *x*_*m*_}, each sample is described with *n* features *F* = {*f*_1_, *f*_2_,…, *f*_*n*_}. One has *F*_1_, *F*_2_⊆*F*. *K*_1_ and *K*_2_ are two kernel matrices induced by *F*_1_ and *F*_2_. *K* is a new kernel function computed with *F*_1_ ∪ *F*_2_. Knowning  *F*_1_, the condition entropy of *F*_2_ is defined as

(8) $$\begin{array}{c} E(K_{1}\;|\;K_{2})=E(K)-E(K_{1}). \end{array}$$

As to the Gaussian kernel, *E*(*K*) ≥ *E*(*K*_1_) and *E*(*K*) ≥ *E*(*K*_2_), so *E*(*K*_1_∣*K*_2_) ≥ 0 and *E*(*K*_2_∣*K*_1_) ≥ 0.

**Definition 4 .**

Given a set of samples *U* = {*x*_1_, *x*_2_,…, *x*_*m*_}, each sample is described with *n* features *F* = {*f*_1_, *f*_2_,…, *f*_*n*_}. One has *F*_1_, *F*_2_⊆*F*. *K*_1_ and *K*_2_ are two kernel matrices induced by *F*_1_ and *F*_2_. *K* is a new kernel function computed with *F*_1_ ∪ *F*_2_. Then the mutual information of *K*_1_ and *K*_2_ is defined as

(9) $$\begin{array}{c} \text{MI}(K_{1},K_{2})=E(K_{1})+E(K_{2})-E(K). \end{array}$$

As to Gaussian kernel, MI(*K*_1_, *K*_2_) = MI(*K*_2_, *K*_1_). If *K*_1_⊆*K*_2_, we have MI(*K*_1_, *K*_2_) = *E*(*K*_2_) and if *K*_2_⊆*K*_1_, we have MI(*K*_1_, *K*_2_) = *E*(*K*_1_).

Please note that if *F*_1_⊆*F*_2_, we have *K*_2_⊆*K*_1_. However, *K*_2_⊆*K*_1_ does not mean *F*_1_⊆*F*_2_.

**Definition 5 .**

Given a set of samples *U* = {*x*_1_, *x*_2_,…, *x*_*m*_}, each sample is described with *n* features *F* = {*f*_1_, *f*_2_,…, *f*_*n*_}. *F*′⊆*F*, *K* is a kernel matrix over *U* in terms of *F*′, and *D* is the kernel matrix computed with the decision. Then the feature significance *F*′ related to the decision is defined as

(10) $$\begin{array}{c} \text{MI}(K,D)=E(K)+E(D)-E(K,D). \end{array}$$

MI(*K*, *D*) measures the importance of feature subset *F*′ in the kernel space to distinguish different classes. It can be understood as a kernelized version of Shannon information entropy, which is widely used feature evaluation selection. In fact, it is easy to derive the equivalence between this entropy function and Shannon entropy in the condition that the attributes are discrete and the matching kernel is used.

Now we show an example in gas turbine fault diagnosis. We collect 3581 samples from two sets of gas turbine systems. 1440 samples are healthy and the others belong to four kinds of faults: load rejection, sensor fault, fuel switching, and salt spray corrosion. The numbers of samples are 45, 588, 71, and 1437, respectively. Thirteen thermometers are installed in the exhaust. According to the approach described above, we form a 40-dimensional vector to represent the state of the exhaust. Obviously, the classification task is not understandable in such high dimensional space. Moreover, some features may be redundant for classification learning, which may confuse the learning algorithm and reduce modeling performance. So it is a key preprocessing step to select the necessary and sufficient subsets.

Here we compare the fuzzy rough set based feature evaluation algorithm with the proposed kernelized mutual information. Fuzzy dependency has been widely discussed and applied in feature selection and attribute reduction these years [26–28]. Fuzzy dependency can be understood as the average distance from the samples and their nearest neighbor belonging to different classes, while the kernelized mutual information reflects the relevance between features and decision in the kernel space.

**Figure 10:**
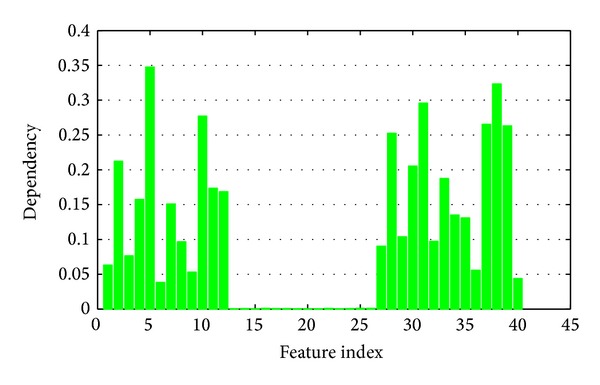
Fuzzy dependency between a single feature and decision.

**Figure 11:**
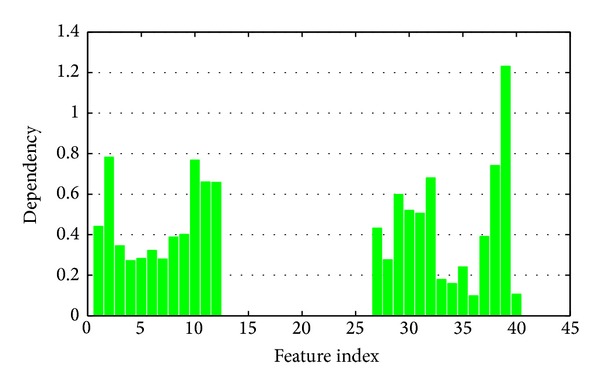
Kernelized mutual information between a single feature and decision.

Comparing Figures 10 and 11, significant difference is obtained. As to fuzzy rough sets, Feature 5 produces the largest dependency and then Feature 38. However, Feature 39 gets the largest mutual information, and Feature 2 is the second one. Thus different feature evaluation functions will lead to completely different results.

**Figure 12:**
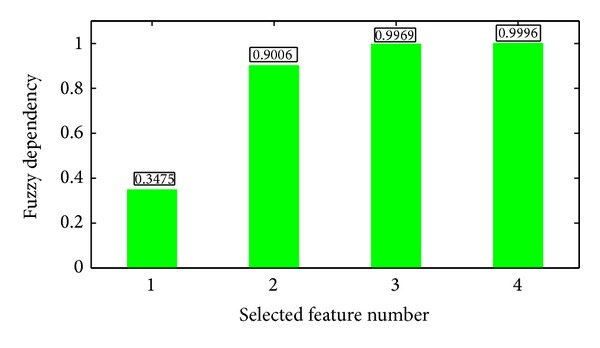
Fuzzy dependency between the selected features and decisions (Features 5, 37, 2, and 3 are selected sequentially).

**Figure 13:**
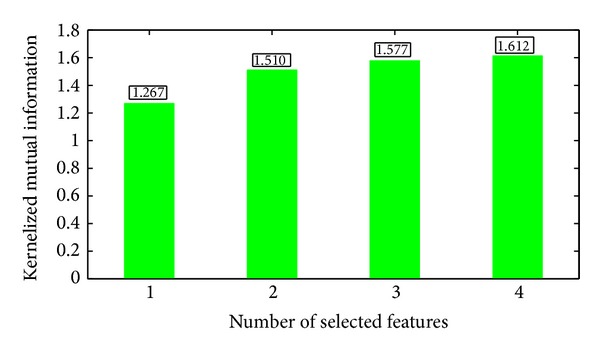
Kernelized mutual information between the selected features and decisions (Features 39, 31, 38, and 40 are selected sequentially).

Figures 10 and 11 present the significance of single features. In applications, we should combine a set of features. Now we consider a greedy search strategy. Starting from an empty set and the best features are added one by one. In each round, we select a feature which produces the largest significance increment with the selected subset. Both fuzzy dependency and kernelized mutual information increase monotonically if new attributes are added. If the selected features are sufficient for classification, these two functions will keep invariant by adding any new attributes. So we can stop the algorithm if the increment of significance is less than a given threshold. The significances of the selected feature subset are shown in Figures 12 and 13, respectively.

**Figure 14:**
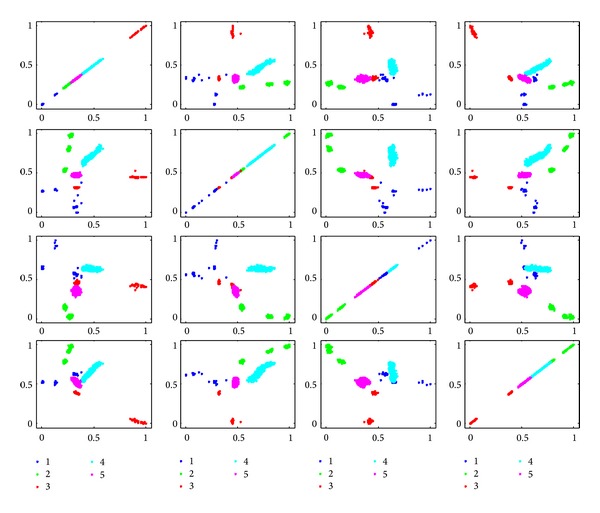
Scatter plots in 2D space expended with feature pairs selected by fuzzy dependency.

**Figure 15:**
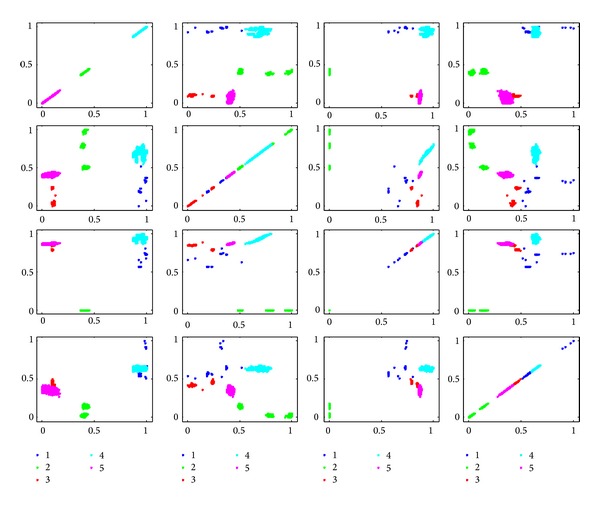
Scatter in 2D space expended with feature pairs selected by kernelized mutual information.

**Figure 16:**
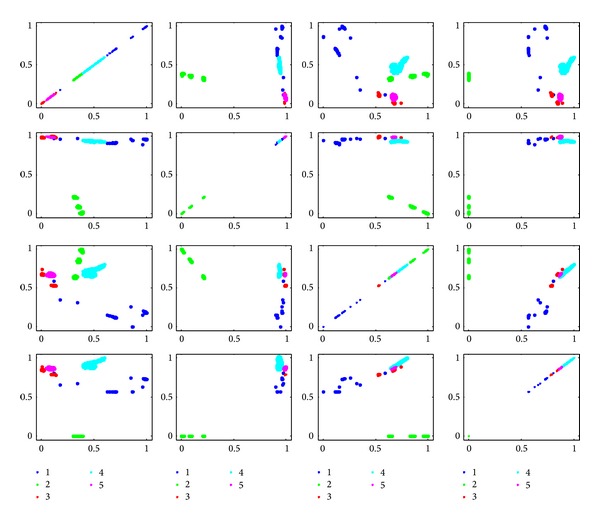
Scatter in 2D space expended with feature pairs selected by Shannon mutual information.

In order to show the effectiveness of the algorithm, we give the scatter plots in 2D spaces, as shown in Figures 14 to 16, which are expended by the feature pairs selected by fuzzy dependency, kernelized mutual information, and Shannon mutual information. As to fuzzy dependency, we select Features 5, 37, 2, and 3. Then there are 4 × 4 = 16 combinations of feature pairs. The subplot in the *i*th row and *j*th column in Figure 14 gives the scatters of samples in 2D space expanded by the *i*th selected feature and the *j*th selected feature.

Observing the 2nd subplots in the first row of Figure 14, we can find that the classification task is nonlinear. The first class is dispersed and the third class is also located at different regions, which leads to the difficulty in learning classification models.

However, in the corresponding subplot of Figure 15, we can see that each class is relatively compact, which leads to a small intraclass distance. Moreover, the samples in five classes can be classified with some linear models, which also bring benefit for learning a simple classification model.

Comparing Figures 15 and 16, we can find that different classes are overlapped in feature spaces selected by Shannon mutual information or get entangled, which leads to the bad classification performance.

## 5. Diagnosis Modeling with Information Entropy Based Decision Tree Algorithm

After selecting the informative features, we now go to classification modeling. There are a great number of learning algorithms for building a classification model. Generalization capability and interpretability are the two most important criteria in evaluating an algorithm. As to fault diagnosis, a domain expert usually accepts a model which is consistent with his common knowledge. Thus, he expects the model is understandable; otherwise, he will not believe the outputs of the model. In addition, if the model is understandable, a domain expert can adapt it according to his prior knowledge, which makes the model suitable for different diagnosis objects.

Decision tree algorithms, including CART [29], ID3 [17], and C4.5 [18], are such techniques for training an understandable classification model. The learned model can be transformed into a set of rules. All these algorithms build a decision tree from training samples. They start from a root node and select one of the features to divide the samples with cuts into different branches according to their feature values. This procedure is interactively conducted until the branch is pure or a stopping criterion is satisfied. The key difference lies in the evaluation function in selecting attributes or cuts. In CART, splitting rules GINI and Twoing are adopted, while ID3 uses information gain and C4.5 takes information gain ratio. Moreover, C4.5 can deal with numerical attributes compared with ID3. Competent performance is usually observed with C4.5 in real-world applications compared with some popular algorithms, including SVM and Baysian net. In this work, we introduce C4.5 to train classification models. The pseudocode of C4.5 is formulated as follows.

 Decision tree algorithm C4.5
 Input: a set of training samples *U* = {*x*_1_, *x*_2_,…, *x*_*m*_} with features *F* = {*f*_1_, *f*_2_,…, *f*_*n*_}
 Stopping criterion *τ*
 Output: decision tree *T*
(1) Check for sample set
(2) For each attribute *f* compute the normalized information gain ratio from splitting on *a*
(3) Let* f_best* be the attribute with the highest normalized information gain
(4) Create a decision node that splits on* f_best*
(5) Recurse on the sublists obtained by splitting on* f*_*best*, and add those nodes as children of node until stopping criterion *τ* is satisfied
(6) Output *T*.

**Figure 17:**
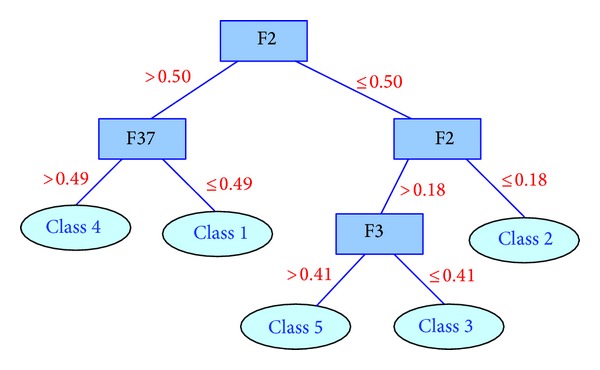
Decision tree trained on the features selected with fuzzy rough sets.

**Figure 18:**
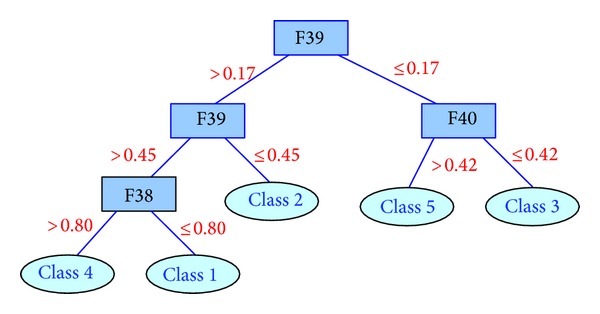
Decision tree trained on the features selected with kernelized mutual information.

We input the data sets into C4.5 and build the following two decision trees. Features 5, 37, 2, and 3 are included in the first dataset, and Features 39, 31, 38, and 40 are selected in the second dataset. The two trees are given in Figures 17 and 18, respectively.

We start from the root node to a leaf node along the branch, and then a piece of rule is extracted from the tree. As to the first tree, we can get five decision rules:

1. if F2 > 0.50 and F37 > 0.49, then the decision is Class 4;
2. if F2 > 0.50 and F37 ≤ 0.49, then the decision is Class 1;
3. if 0.18 < F2 ≤ 0.50 and F3 > 0.41, then the decision is Class 5;
4. if 0.18 < F2 ≤ 0.50 and F3 ≤ 0.41, then the decision is Class 3;
5. if F2 ≤ 0.18, then the decision is Class 2.

As to the second decision tree, we can also obtain some rules as

1. if F39 > 0.45 and F38 > 0.80, then the decision is Class 4;
2. if F39 > 0.45 and F38 ≤ 0.80, then the decision is Class 1;
3. if 0.17 < F39 ≤ 0.45, then the decision is Class 2;
4. if F39 ≤ 0.17 and F40 > 0.42, then the decision is Class 5;
5. if F39 ≤ 0.17, and F40 ≤ 0.42, then the decision is Class 3.

We can see the derived decision trees are rather simple and each can extract five pieces of rules. It is very easy for domain experts to understand the rules and even revise the rules. As the classification task is a little simple, the accuracy of each model is high to 97%. As new samples and faults are recorded by the system, more and more complex tasks may be stored. In that case, the model may become more and more complex.

## 6. Conclusions and Future Works

Automatic fault detection and diagnosis are highly desirable in some industries, such as offshore oil well drilling platforms, for such systems are self-monitoring without man on duty. In this work, we design an intelligent abnormality detection and fault recognition technique for the exhaust system of gas turbines based on information entropy, which is used in measuring the uniformity of exhaust temperatures, evaluating the significance of features in kernel spaces, and selecting splitting nodes for constructing decision trees. The main contributions of the work are two parts. First, we introduce the entropy function to measure the uniformity of exhaust temperatures. The measurement is easy to compute and understand. Numerical experiments also show its effectiveness. Second, we extend Shannon entropy for evaluating the significance of attributes in kernelized feature spaces. We compute the relevance between a kernel matrix induced with a set of attributes and the matrix computed with the decision variable. Some numerical experiments are also presented. Good results are derived.

Although this work gives an effective framework for automatic fault detection and recognition, the proposed technique is not tested on large-scale real tasks. We have developed a remote state monitoring and fault diagnosis system. Large scale data are flooding into the center. In the future, we will improve these techniques and develop a reliable diagnosis system.
